# Single-cell analyses reveal the dynamic functions of Itgb2^+^ microglia subclusters at different stages of cerebral ischemia-reperfusion injury in transient middle cerebral occlusion mice model

**DOI:** 10.3389/fimmu.2023.1114663

**Published:** 2023-03-30

**Authors:** Fanning Zeng, Jun Cao, Zexuan Hong, Yujun Liu, Jie Hao, Zaisheng Qin, Xin Zou, Tao Tao

**Affiliations:** ^1^ Department of Anesthesiology, Central People’s Hospital of Zhanjiang, Zhanjiang, Guangdong, China; ^2^ Department of Anesthesiology, Nanfang Hospital, Southern Medical University, Guangzhou, Guangdong, China; ^3^ Department of Anesthesiology, Affiliated Shenzhen Maternity and Child Healthcare Hospital, Southern Medical University, Shenzhen, Guangdong, China; ^4^ Center for Tumor Diagnosis and Therapy, Jinshan Hospital, Fudan University, Shanghai, China; ^5^ Institute of Clinical Science, Zhongshan Hospital, Fudan University, Shanghai, China; ^6^ Department of Pathology, Jinshan Hospital, Fudan University, Shanghai, China

**Keywords:** single cell RNA-seq, Itgb2, scSTAR, microglia, cerebral ischemia-reperfusion injury

## Abstract

**Introduction:**

The underlying pathophysiological mechanisms of cerebral ischemia reperfusion injury (CIRI) is intricate, and current studies suggest that neuron, astrocyte, microglia, endothelial cell, and pericyte all have different phenotypic changes of specific cell types after ischemic stroke. And microglia account for the largest proportion after CIRI. Previous transcriptomic studies of ischemic stroke have typically focused on the 24 hours after CIRI, obscuring the dynamics of cellular subclusters throughout the disease process. Therefore, traditional methods for identifying cell types and their subclusters may not be sufficient to fully unveil the complexity of single-cell transcriptional profile dynamics caused by an ischemic stroke.

**Methods:**

In this study, to explore the dynamic transcriptional profile of single cells after CIRI, we used single-cell State Transition Across-samples of RNA-seq data (scSTAR), a new bioinformatics method, to analyze the single-cell transcriptional profile of day 1, 3, and 7 of transient middle cerebral artery occlusion (tMCAO) mice. Combining our bulk RNA sequences and proteomics data, we found the importance of the integrin beta 2 (Itgb2) gene in post-modeling. And microglia of Itgb2+ and Itgb2- were clustered by the scSTAR method. Finally, the functions of the subpopulations were defined by Matescape, and three different time points after tMCAO were found to exhibit specific functions.

**Results:**

Our analysis revealed a dynamic transcriptional profile of single cells in microglia after tMCAO and explored the important role of Itgb2 contributed to microglia by combined transcriptomics and proteomics analysis after modeling. Our further analysis revealed that the Itgb2+ microglia subcluster was mainly involved in energy metabolism, cell cycle, angiogenesis, neuronal myelin formation, and repair at 1, 3, and 7 days after tMCAO, respectively.

**Discussion:**

Our results suggested that Itgb2+ microglia act as a time-specific multifunctional immunomodulatory subcluster during CIRI, and the underlying mechanisms remain to be further investigated.

## Introduction

Stroke is one of the major causes of death and disability in adults (1, 2). Approximately 87% of strokes are ischemic strokes, characterized by the reduced blood supply to the brain (3). Currently, the main strategy for the treatment of ischemic stroke is to revascularize the infarcted vessels and restore the blood supply to the ischemic area as soon as possible, such as pharmacological thrombolysis and mechanical endovascular thrombus extraction (4). Nevertheless, restoring blood flow to the ischemic region can subsequently exacerbate brain injury, which is known as cerebral ischemic-reperfusion injury (CIRI). The underlying pathophysiological mechanism of CIRI remains intricate and complex, including metabolism, excitotoxicity, and inflammation which could result in various forms of cell death, including necrosis, apoptosis, and pyroptosis (5, 6). The current study demonstrates that neurons, astrocytes, microglia, endothelial cells, and pericytes all have different phenotypic changes in specific cell types after ischemic stroke (7, 8). And after cerebral blood flow disruption, microglia accounted for the largest proportion of cell numbers after transient middle cerebral artery occlusion (tMCAO) (8). Previous transcriptomic studies of ischemic stroke have typically focused on the 24 hours after ischemia-reperfusion, obscuring the impact of the disease progression process. Different cellular subclusters at the time points are essential throughout the disease.

Single-cell RNA sequencing (9) as an outstanding high-throughput sequencing technology could facilitate to explore the cellular and molecular changes during cerebral ischemic reperfusion injury. In the previous study, Shi et al. found that Treg cells enriched in the ischemic brain through single cell RNA-sequence (scRNA-seq), and demonstrated that Treg cells could drive microglia to regulate the differentiation of oligodendrocyte precursor cells, which ultimately influenced the long-term outcomes after stroke (10). Another scRNA-seq research (7) of cerebral ischemia revealed that immune cells (including microglia, monocytes and macrophages), vasculature cells, and ependymal cells may play important roles during neuroinflammation induced by ischemic-reperfusion injury. More recently, Li and his colleagues (11) depicted the landscape of immune cells after different time points of cerebral ischemic stroke in aged mice through scRNA-seq during 24 hours after tMCAO. Their results identified a stroke-specific microglia subtype. Taken together, these cerebral ischemia scRNA-seq studies indicated that immune cells, especially microglia, act as vital roles during cerebral reperfusion injury by regulating neuroimmune and neuroinflammation. And their results provided a novel avenue for cerebral ischemia research. However, as depicted by the above studies, the transcriptional profiles of different cell clusters and their subclusters are varied dynamically during the pathophysiological process of cerebral ischemic-reperfusion injury. Therefore, the traditional methods to identify the cell types and their subclusters may not be full enough to exhibit the complexity of single-cell transcriptional profile dynamics during ischemic stroke.

In this study, to determine the single-cell dynamic transcriptional profiles after cerebral reperfusion injury, we adopted single-cell State Transition Across-samples of RNA-seq data (scSTAR) (12) to analyze the single cell transcriptional profiles from tMCAO mouse brains on day 1,3 and 7. Most of the current single-cell analysis techniques are based on directly comparing changes in cell populations after intervention and modeling. Although such comparisons can reveal changes in the number of cells in the cell clusters or the appearance of new cell clusters after intervention and modeling which can be used to speculate on disease mechanisms or molecular biological functions, existing analyses may not be sensitive to molecular function alterations as those changes may be masked by stronger cell-cell heterogeneities, such as cell subtypes. The existing analysis processes lose the detailed dynamics changes after intervention or modeling. scSTAR (12) can capture such trivial but significant variations by comparing the constructed real-virtual cell pairs, which can screen the variations irrelevant to the biological problem studied because the differences between the real and virtual cell pairs are only caused by the designed intervention. Based on scSTAR analysis, we determined the molecular dynamics features of cell subclusters changed by tMCAO at different time points, which facilitate us to explore and understand the dynamic transcription variation during CIRI. Then, combined with our bulk RNA-sequence data and proteomics data, we found Itgb2^+^ subclusters of microglia exhibited particular functions on day 1,3 and 7 after the onset of reperfusion. Overall, our study screened novel subclusters of microglia which may have versatile functions during cerebral reperfusion injury based on scSTAR bioinformatic analysis. Besides, we also provide a scRNA-seq data set of cerebral reperfusion injury for further study.

## Materials and methods

### Animals

Male C57/BL6 mice (8-10 weeks old, 22 ± 2 g, Specific Pathogen Free) were purchased from the Experimental Animal Center of Southern Medical University (Guangzhou, China). Animals were kept in cages under standard light and dark cycle conditions (12 hrs:12 hrs, temperature 25 °C) with free access to food and water. In addition, cages were cleaned regularly. All animal studies were conducted in accordance with protocols and guidelines approved by the Institutional Animal Ethical Care Committee of the Southern Medical University Laboratory Animal Center.

### Transient middle cerebral artery occlusion model establishment

The modeling of temporary middle cerebral artery occlusion has been described in our previous article (13). Briefly, mice were anesthetized with continuous inhalation of sevoflurane (2%-5%); the inner and outer muscles of the sternocleidomastoid muscle were separated to expose and isolate the right common, external, and internal carotid arteries. Subsequently, the superior thyroid and occipital arteries were separated and cauterized using a preheated electrocautery to prevent bleeding. The model was established by inserting a monofilament (approximately 2 cm) from the external carotid artery to the middle cerebral artery, avoiding the pterygopalatine artery. After the monofilament was inserted for 1.5 hours of ischemia, the monofilament was gently pulled out to form reperfusion. The wound was disinfected with iodine and sutured. Mice in the sham group adopted the same procedure except for the MCA occlusion (14). Cerebral blood flow (CBF) was measured continuously in mice before, during and after MCAO surgery by applying a laser Doppler flowmeter (Periflux System 5000, Perimed, Järfälla, Sweden). A decrease in CBF in the MCAA region ≥ 80% of baseline after MCAO surgery and recovery of CBF ≥ 70% of baseline within 10 minutes after dismantling were considered successful in inducing brain I/R injury; otherwise, mice were excluded (15). After the behavioral tests on 1, 3, and 7 days after modeling, the mouse brain samples from the ipsilateral hemisphere were taken for single–cell isolation and extraction.

### Infarct volume analysis

TTC (2,3,5-Triphenyltetrazolium chloride) staining was used to reflect cerebral infarction as a percentage of brain volume. The mice were anesthetized and the integral brains were quickly obtained and cut into 2 mm tissue slices, then stained with 2% TTC for 5 minutes and soaked in 4% formaldehyde for 6 hours. The brain slices were arranged in order and photographed. The red area indicated no infarction; the white area indicated infarction.

### Rotarod test

Sensorimotor functions were accessed by the rotarod test after tMCAO. All mice were trained for 2 days before the model establishment (each mouse was tested twice and the speed of rotation was 5 rpm for 10 minutes). During rotarod testing, the speed of rotation was accelerated from 5 to 15 rpm over 60 seconds with a testing period cut-off of 300 seconds for each trial and 2 total trials performed. The fall latency of each mouse was recorded and averaged. The experimenter was blinded to the treatments given to each mouse (13).

### Neurological scoring

The neurological deficit (ND) score was evaluated by a blinded investigator after 1, 3, and 7 days of reperfusion as previously described. The scoring system was based on the following criteria: (1) no neurological deficit (0 points); (2) forelimb weakness and torso turning to the ipsilateral side when held by the tail (1 point); (3) circling to affected side (2 points); (4) unable to bear weight on the affected side (3 points); and (4) no spontaneous locomotor activity or barrel rolling (4 points).

### scRNA-seq data generation

Beads with unique molecular identifiers (UMI) and cell barcodes were loaded close to saturation so that each cell was paired with a bead in a Gel Beads-in-emulsion (GEM). After exposure to cell lysis buffer, polyadenylated RNA molecules hybridized into the beads. Beads were retrieved into a single tube for reverse transcription. On cDNA synthesis, each cDNA molecule was tagged on the 5’end (that is, the 3’end of a messenger RNA transcript) with UMI and cell label indicating its cell of origin. Briefly, 10× beads were then subject to second-strand cDNA synthesis, adaptor ligation, and universal amplification. Sequencing libraries were prepared using randomly interrupted whole-transcriptome amplification products to enrich the 3’end of the transcripts linked with the cell barcode and UMI. All the remaining procedures including the library construction were performed according to the standard manufacturer’s protocol (CG000206 RevD). Sequencing libraries were quantified using a High Sensitivity DNA Chip (Agilent) on a Bioanalyzer 2100 and the Qubit High Sensitivity DNA Assay (Thermo Fisher Scientific). The libraries were sequenced on NovaSeq6000 (Illumina) using 2×150 chemistry.

### scRNA-seq data processing, quality control and integration

The FASTQ files were generated by using the 10× Genomics Cell Ranger toolkit (v.3.0.1), which were extracted barcodes and UMI, filtered and mapped reads to the GRCh38 reference genome, and obtained a matrix containing normalized gene counts versus cells per sample. Then we further analyzed these outputs using Seurat (v.4.1.2) for quality control and downstream analysis with default parameters, unless otherwise indicated. We removed out low-quality cells according to the following quality control procedures: (1) cells with less than 200 genes or more than 3000 genes and genes expressed in less than 3 cells were removed. (2) over 10% of mitochondrial-derived UMI counts were filtered out. After quality control, the datasets were log-normalized. The top 2000 highly variable genes (HVGs) were chosen for canonical correlation analysis and the first 30 reduced dimensions were adopted for integration anchors. Finally, a total of 53484 single cells were applied in downstream analyses, including 16345, 11056, 16246 and 9837 single cells on postoperative days 1, 3 and 7 and sham surgery groups, respectively.

### Dimensionality reduction, clustering, annotation and visualization

Then the data were scaled using the ScaleData function. Principal components analysis (PCA) was performed, and we selected the top 30 PCs for clustering cells with a resolution parameter of 1.0. The same PCs were used to generate the Uniform manifold approximation and projection (UMAP). Each cluster was annotated based on high expression of known marker genes and visualized by FeaturePlot function. Cldn5, Ly6c1, Ly6a, Itm2a (Endothelial Cells); Cldn11, Mag, Opalin, Ermn, Mog (Oligodendrocytes); Acta2, Des, Tpm2, Filip1l (Vascular Smooth-muscle Cells); Ttr, Clic6, Sostdc1, Car12, Prlr, Htr2c (Choroid plexus cells); VKcnj8, Atp13a5, Anpep, Abcc9, Cd248 (Pericytes); Hexb, C1qa, Tmem119, GRP34, OLFML3 (Microglia); Rtn1, Sox4 (Neuron); F3, Gja1, Slc1a3, Gjb6(Astrocyte); Calml4, Chchd10, Clu (Ependymal); Olig1, Olig2, Pdgfra, Tnr, C1ql1, Matn4 (Oligodendrocyte progenitor cells); Cd8a, Klrd1, Cd4 (T cells); Trem1, Mmp8 (Neutrophil); Cd14, Cd86 (Monocyte); Cd19, Cd79a (B cells).

### scSTAR analysis

scSTAR (12), which uses a supervised machine learning algorithm, partial least square (PLS), profiles the differences between two biological feature spaces by maximizing the covariance of the global characteristics. Based on the PLS model, each real cell in one condition can be virtually projected to the counterpart space. The differences between each real-virtual cell pair represent the state transition of the cell driven by the intervention studied. First, the normalized data were categorized into two groups, which represented different biological conditions of the study. The group categorization criteria varied depending on the biological problem of interest. We used the OGFSC (16) package to filter genes on the normalized data, and a dual PLS model was then applied to extract the differences between the two groups. Thus, all variation components that are not related to the signal of interest are excluded and the remaining amplitude represents the differential expression (DE) genes between the two groups. The heterogeneities between cells indicate the diverse dynamic patterns of those cells when conditions change. Finally, all cells from both groups were clustered using the Seurat R package (v.4.1.2) based on processed data. Then, the Seurat ‘FindAllMarkers’ function was applied to identify the marker genes in each cluster. A hypergeometric test is then applied to assign cell clusters to biological conditions. We defined the marker genes of one cluster as upregulated in one condition if the cells from the condition dominated the cluster, as indicated by the hypergeometric test p<0.05. On some occasions, a cell cluster may be associated with no condition, which implies that this cluster of cells tends to be stable.

### Co-analysis of RNA and proteome sequence

Proteome sequencing data were obtained from the tMCAO model in male C57/BL6 mice (8-10 weeks old) on the 3rd postoperative day. RNA-seq (17) was obtained from the tMCAO model in male C57/BL6 mice (8-10 weeks old) 24 h after modeling. Based on our published data from RNA (17) and proteomics sequencing data. Differential genes(|log2 (fold change)| ≥1, p<0.05) in both sequence data were merged. The 44 shared differential genes were obtained and visualized by Graphpad 8.4.2(GraphPad Software, San Diego, CA, USA.) and Cytoscape v. 3.9.1.

### Analyze network

The list of genes in the intersection was imported into the STRING11.5 platform (https://cn.string-db.org/) to determine the protein interaction between the targets. The species was selected as *Mus musculus*, and the “medium confidence” was set to 0.4 to more comprehensively display the relationship between the target genes. Then, the results of the protein-protein interaction (PPI) network analysis were imported into Cytoscape 3.9.1 software in the format of the tsv text, and the Network Analyzer plug-in was used for topology analysis to highlight the importance of target genes and eliminate the target with the node degree of zero. The importance of each gene (node in the network) is determined by the median of degree centrality, medium centrality and compactness centrality was used as the chi-square value. The genes with larger nodes and darker colors perform more important biological functions.

### Metascape analyze

The FindAllMarker function in the Seurat package was used to obtain the differentially expressed genes in clusters using the Wilcoxon rank-sum test. A Bonferroni FDR correction of less than 0.05 was used as a cutoff for identifying statistically significant differentially expressed genes (DEGs). The DEGs of sc_S1, 3, 4, and 5 of the Itgb2^+^ scSTAR analysis were used for the Matescape analysis (https://metascape.org/) (18). The DEG lists of each cluster were provided respectively to the Matescape. M. musculus was selected as the species for input and analysis. The express analysis was selected. Metascape was used to enrich biological processes as well as to cluster biological processes with similar functions and then connect these enriched clusters according to the MCODE algorithm. Graphs of the protein interaction networks were visualized in Cytoscape v3.9.1.

### Kyoto Encyclopedia of Genes and Genome (KEGG) analyze

In order to clarify the interactions of Itgb2 and protein in function-related MCODE, the protein-protein interaction (PPI) lists of MCODE-x (1,2,3…, n) together with Itgb2 were used to perform KEGG enrichment analysis *via* GSEA (http://www.gsea-msigdb.org/gsea/index.jsp), respectively. Gene sets with a significance level of false discovery rate (FDR) of < 0.05 were considered significant. Visualization of KEGG pathways was done by Biorender (https://app.biorender.com/).

## Result

### Identification of major brain cell types after tMCAO

**Figure 1:**
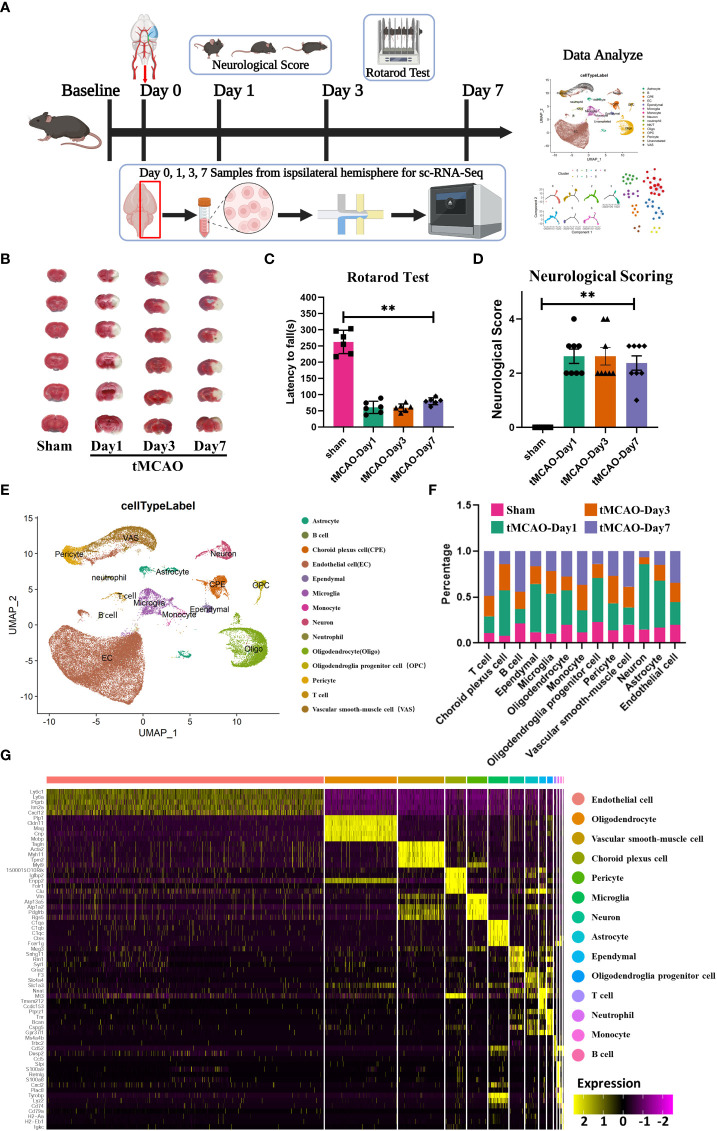
Identification of major brain cell types after tMCAO. **(A)** Flow chart of the experiment. **(B)** TTC staining of mouse brain sections after tMCAO. **(C)** Rotarod test and **(D)** Neurological Score after tMCAO. **: p < 0.001 (two-way analysis of variance followed by Dunnett's multiple comparisons tests) **(E)** UMAP graph of single-cell RNA sequencing subpopulations of the brain sample after tMCAO or sham. **(F)** Cell population ratios at each time point. **(G)** Heatmap of top 5 genes for each cell population.

To investigate cell heterogeneity after ischemic brain injury, we performed behavioral tests on days 1, 3, and 7 after tMCAO as well as we analyzed the mRNA transcripts of single cells extracted from the brain. Single cells were extracted from the brain using the 10× Genomics technology (**Figure 1A**). After tMCAO, we used TTC staining, neurobehavioral test, and neurological scores to assess brain injury in mice. As shown in **Figure 1B**, the mice of the tMCAO group had larger infarct volumes than the sham group. The rotation duration of the sham group (262.5 ± 65.6 s) was longer than the tMCAO group (60.7 ± 34.7 s, 59.5 ± 21.4 s and 79.5 ± 19.6 s) on 1, 3 and 7 days after surgery, respectively (**Figure 1C**). Additionally, the neurological functions of tMCAO mice were impaired significantly compared with the sham mice (**Figure 1D**). As depicted in **Figure 1E**, all single cells were projected on a UMAP plot after batch-effect correction using Seurat and each cell from either tMCAO or sham animals was distinctly assigned to different populations. And we presented the top 5 genes of each cell type in the heatmap (**Figure 1G**). We performed a percentage of cell counts for the defined types of cells. The percentage of microglia on day 1, day 3 and day7 after surgery(43.54%, 24.32% and 21.75%)was significantly higher than that of the sham group (10.40%) (**Figure 1F**). We analyzed intercellular interactions between microglia-immune cells and neuron-immune cells and showed the number of receptor-ligand pairs with heatmaps and chord-chart (**Supplementary Figures 1A, B**). We found abundant receptor-ligand pairs between microglia and immune cells, suggesting that microglia play a central role in the immune response. Also, the number of receptor-ligand pairs between microglia and neurons was the highest among immune cells at 68, suggesting that microglia have the opportunity to influence neuronal function by acting on neurons. The detailed receptor-ligand pairs are shown in **Supplementary Figures 2**, **3**. The results above suggested the importance of microglia after tMCAO modeling.

### Overview of scSTAR analysis of microglia

**Figure 2:**
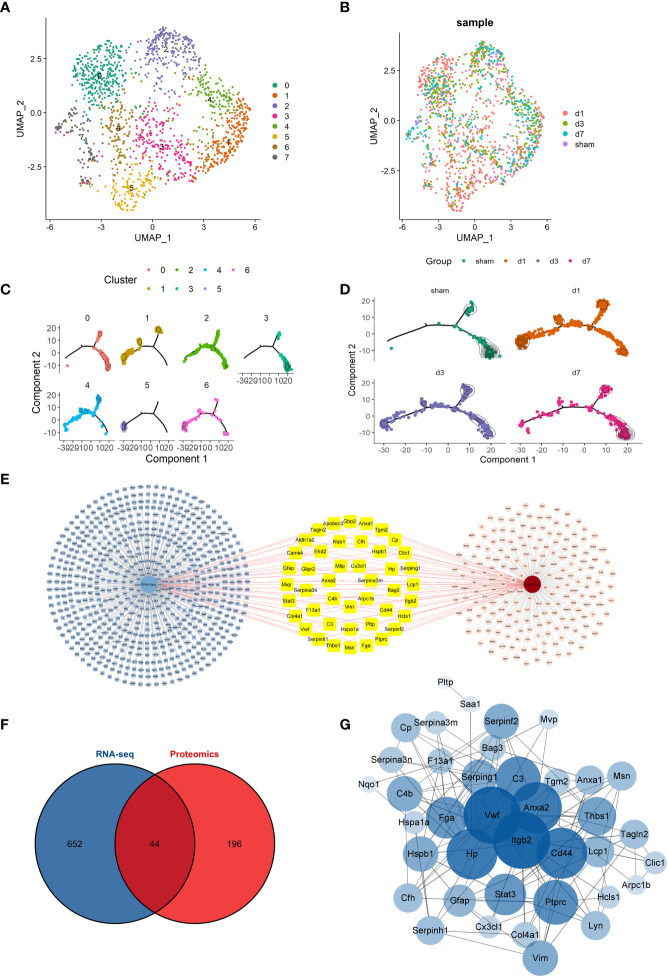
Overview of conventional scRNA-seq analysis and scSTAR analysis of microglia **(A, B)** UMAP of the microglia subcluster and the distribution of cells at time points by the conventional analysis method. **(C, D)** Trajectory analysis of the microglia subcluster and the distribution of cells at time points by scSTAR. **(E)** Details of 44 differentially expressed genes co-expressed in RNA-Seq and proteomic. **(F)** A Venn plot of 44 DEGs was both in RNA-Seq and proteomic differentially expressed genes or proteins. **(G)** Network Analyzer of 44 shared DEGs.

We next analyzed and demonstrated the changes in cellular dynamics of microglia at different time points after tMCAO modeling. We first used the conventional analysis method to sort the microglia. Microglia were divided into 8 subclusters and the distribution of cells at each time point is presented in **Figures 2A, B** in the form of UMAP. We performed a functional enrichment analysis of microglia subcluster. Some subclusters of microglia had specific functions. Cluster1 was involved in inflammation, Cluster3 in metabolism-related processes, and Cluster6, 7 in glial cell differentiation and gliogenesis, as well as neurogenesis and synapses (**Supplementary Figure 4A**). The top 5 genes for each subcluster were presented in the heatmap (**Supplementary Figure 4B**). To clarify the relationship of microglia function related to time points, we next performed the scSTAR analysis. Based on dynamic analysis, we reclassified microglia into 7 subclusters and also analyzed cells at different time points (**Figures 2C, D**). We found that there was a clear tendency for the cells to aggregate from the right to the left branch after tMCAO (**Figure 2D**) and this change gradually recovered over time. Based on the scSTAR subclusters, the distribution of cluster 4 was similar to that of the Sham group, while the distribution of clusters 5 and 6 was consistent with the dynamic changes after modeling. This suggested that different microglia subclusters might function during the time points.

We then intersected the RNA-seq differentially expressed genes and the differentially expressed proteins of the proteomic of the tMCAO model. A total of 44 genes were both in RNA-Seq and proteomic differentially expressed genes or proteins (**Figure 2F**). The 44 genes were shown in detail in **Figure 2E**. Then, the 44 genes were analyzed by STRING and the Network Analyzer plug in of Cytoscape 3.9.1 software to obtain the genes’ PPI network and topological characteristics. The network details are shown in **Figure 2G**. Genes with the top 10 highest degrees of freedom were integrin beta 2(Itgb2), Von Willebrand factor (Vwf), annexin A2 (Anxa2), CD44 antigen (Cd44), haptoglobin (Hp), complement component 3 (C3), protein tyrosine phosphatase, receptor type, C (Ptprc), fibrinogen alpha chain (Fga), signal transducer and activator of transcription 3 (Stat3), and signal transducer and activator of transcription 3 (Thbs1). Itgb2, the most important gene, was selected for further scSTAR analysis to determine Itgb2 function in microglia at different time points.

### scSTAR of itgb2^+^ cell clusters in groups on 1, 3 and 7 days after tMCAO

**Figure 3:**
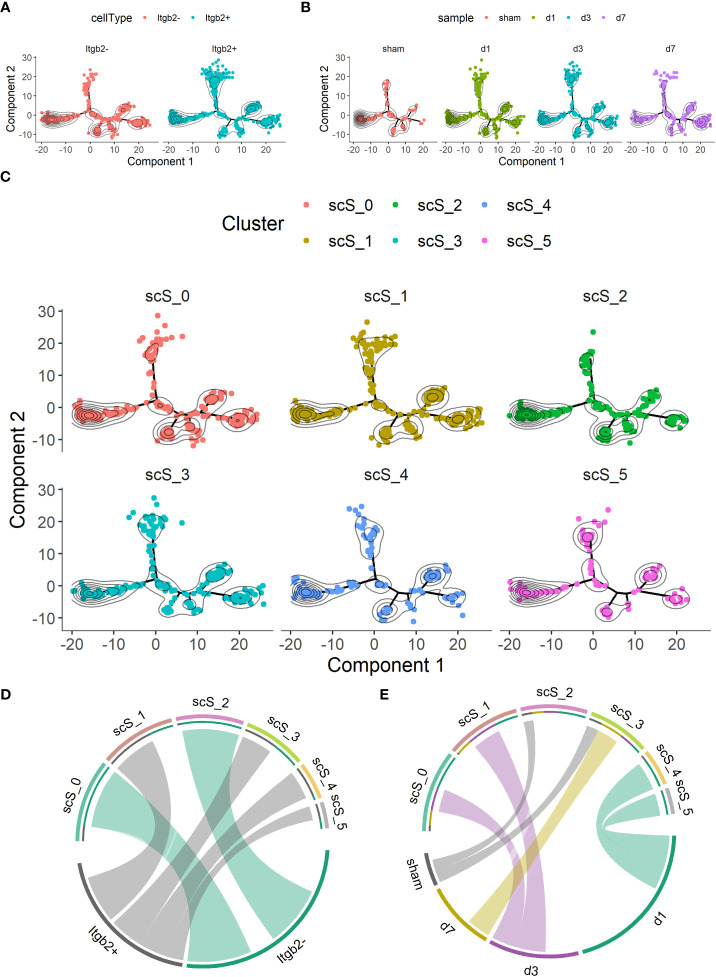
scSTAR of itgb2^+^ microglia subclusters in groups on day 1, 3 and 7 after tMCAO **(A)** Trajectory analysis of the Itgb2^+^ microglia subcluster by scSTAR. **(B)** The distribution of cells at time points on trajectory by scSTAR. **(C)** The trajectory of 6 microglia subclusters by scSTAR. **(D)** The relationship with Itgb2 expression pattern and subclusters by scSTAR after tMCAO via chord-charts. **(E)** The relationship with Itgb2 expression pattern and time points after tMCAO *via* chord-charts.

Next, we performed a scSTAR analysis for Itgb2. First, all microglia were divided into Itgb2^+^ and Itgb2^-^ clusters. Trajectory analysis showed that the Itgb2^+^ cluster was more concentrated on the upper and right side than the Itgb2^-^ cluster (**Figure 3A**). It could be seen in the trajectory of the time points that the subclusters were aggregated from the left branch to the uppermost and rightmost ones after tMCAO modeling and these changes were dynamic. These trajectory analyses indicated that Itgb2^+^ microglia may play an important role after tMCAO (**Figure 3B**). Then, the microglia were further populated into 6 microglia subclusters. We used to chord-charts clarify the further relationship between subclusters with Itgb2^+^ cluster and time points after tMCAO, respectively (**Figure 3C**). Based on the Itgb2 expression, the Itgb2^+^ was correspondent to scS_1, 3, 4, 5 microglia subclusters, while Itgb2^-^ was correspondent to scS_0 and 2 (**Figure 3D**). From the time points, the sham group corresponded to the microglia subclusters of sc_S2 and sc_S3, the day1 group corresponded to the microglia subclusters of sc_S4 and sc_S5, the day3 group corresponded to the microglia subclusters of sc_S0 and sc_S1, the day7 group corresponded to the microglia subcluster of sc_S3 (**Figure 3E**). These results suggested that the different Itgb2^+^ microglia subclusters corresponded to different time points and subclusters may have time-specific molecular functions. Subsequently, these Itgb2^+^ microglia subclusters were further analyzed.

### Functional enrichment of the Itgb2+ cell cluster on day 1 after tMCAO

**Figure 4:**
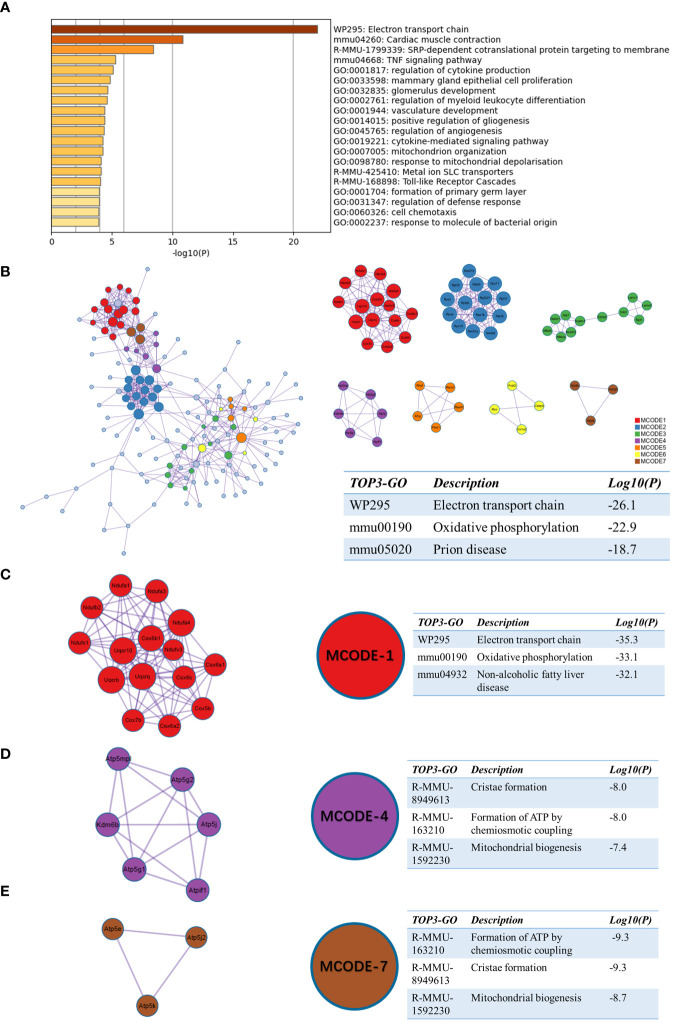
Functional enrichment of the sc_S5 on day 1 after tMCAO. **(A)** The top 20 enriched terms of the sc_S5 on day 1 by Metascape, colored by p-values. **(B)** Protein-protein interaction network and all MCODE components identified of the sc_S5 on day 1. **(C–E)** Representative MCODEs with correlation to enrichment analysis of the sc_S5.

We next performed a functional enrichment analysis of sc_S4 and sc_S5 associated with day 1 after tMCAO by Matescape, respectively. The top 1 enriched term of the sc_S5 was “electron transport chain” (logP=-21.97891176) (**Figure 4A**). In GO/KEGG terminology, WikiPathways, Reactome Gene Sets, enriched terms such as “oxidative phosphorylation”, “ATP metabolic processes” and “respiratory electron transport” were identified (**Supplementary Table 1**). These results indicated that sc_S5 might play an important role in energy metabolism during day 1 after tMCAO. To clarify the specific genes involved in energy metabolism, we applied a PPI network and MCODE components to identify the DEGs in sc_S5. The three best-scoring terms by p-value are shown in the table to the right of the corresponding network diagram in **Figure 4B**. The top 2 of the three best-scoring terms were “Electron transport chain” and “Oxidative phosphorylation”, which further supported our speculation. The following MCODE analysis screened out three groups of genes involved in a network of protein-protein interactions related to energy metabolism. The three best-scoring terms by p-value for functional descriptions and the corresponding specific genes in the MCODE group are shown in **Figures 4C–E**. The above results suggested that the Itgb2^+^ sc_S5 microglia subcluster may closely relate to energy metabolism on the first day after tMCAO.

**Figure 5:**
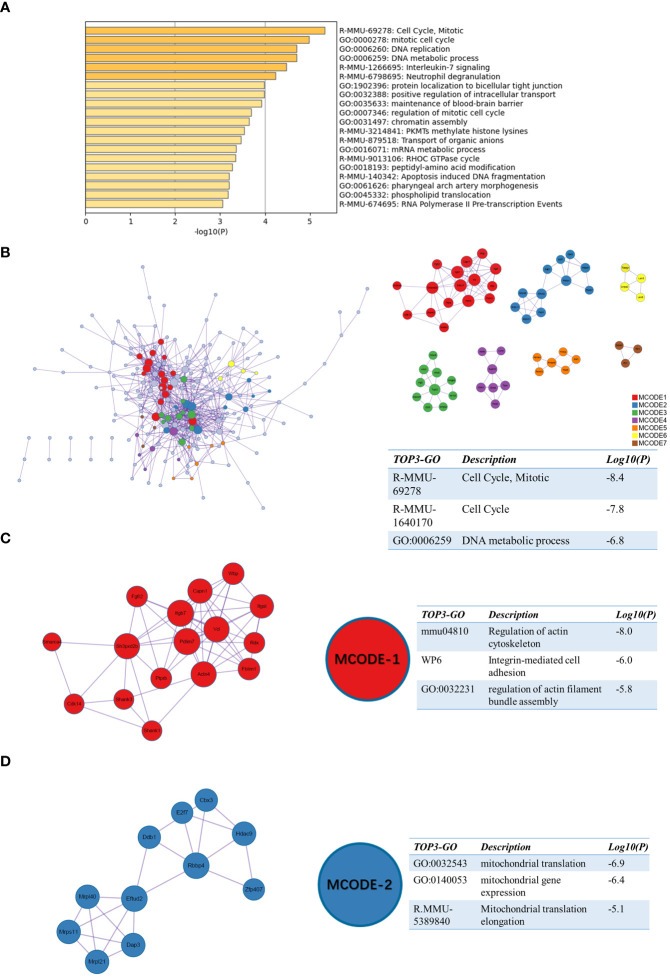
Functional enrichment of the sc_S4 on day 1 after tMCAO. **(A)** The top 20 enriched terms of the sc_S4 on day 1 by Metascape, colored by p-values. **(B)** Protein-protein interaction network and all MCODE components identified of the sc_S4 on day 1. **(C, D)** Representative MCODEs with correlation to enrichment analysis of the sc_S4.

We also performed enrichment analysis on sc_S4 by Matescape. Unlike sc_S5, the functions of sc_S4 are more enriched in “Cell Cycle, Mitotic”, “Synthesis of DNA” and “mRNA metabolic process” (**Figure 5A**). The three best-scoring terms by the p-value of the PPI network were “Cell Cycle, Mitotic”, “Cell Cycle” and “DNA metabolic process” (**Figure 5B**). In the MCODE analysis, the three highest scoring terms in the MODE-1 group included “Integrin-mediated cell adhesion” suggesting the important role of Itgb2 as one of the subunits of the integrin family in DNA and RNA synthesis (**Figure 5C**). The three best-scoring terms in the MODE-2 group all involved mitochondrial translation and gene expression (**Figure 5D**) and these corresponded to the functions in sc_S5. The sc_S4 of Itgb2^+^ cells were mainly involved in the cell cycle and RNA splicing process, which we inferred was inextricably related to the proliferation and differentiation of inflammatory cells and the various types of cells performing their functions in the early phase of ischemic stroke.

### The sc_S1 on day 3 after tMCAO involved in angiogenesis

**Figure 6:**
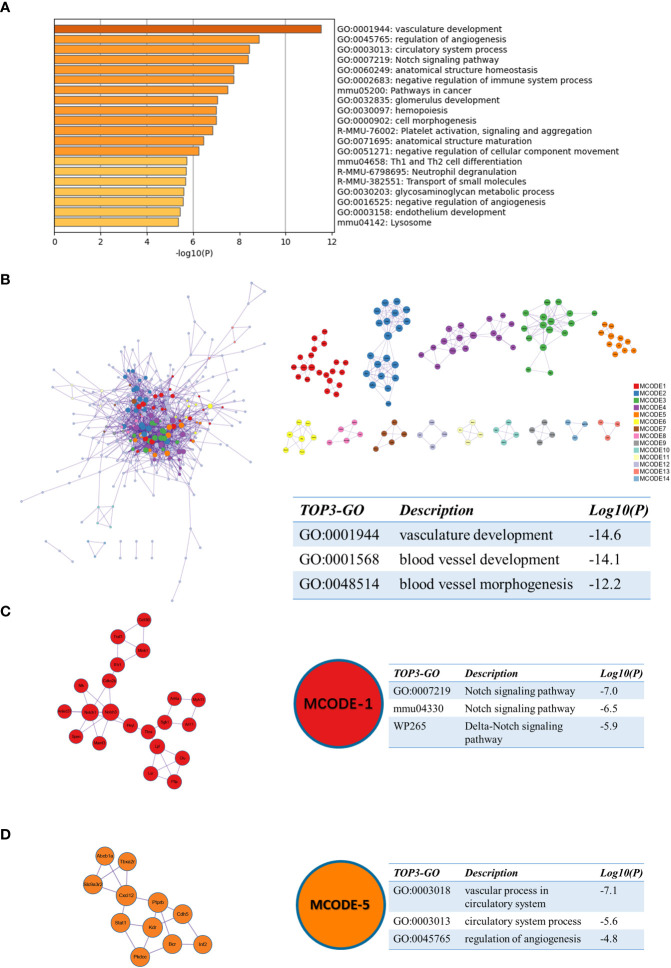
Functional enrichment of the sc_S1 on day 3 after tMCAO. **(A)** The top 20 enriched terms of the sc_S1 on day 3 by Metascape, colored by p-values. **(B)** Protein-protein interaction network and all MCODE components identified of the sc_S1 on day 3. **(C, D)** Representative MCODEs with correlation to enrichment analysis of the sc_S1.

Similarly, we performed enrichment analysis by Metascape for sc_S1 of day 3 after tMCAO. The top 3 enriched terms were “vasculature development”, “regulation of angiogenesis” and “circulatory system process” (**Figure 6A**). The three best-scoring terms by the p-value of the PPI network were “vasculature development”, “blood vessel development” and “blood vessel morphogenesis” (**Figure 6B**). In the analysis of MCODE, we identified two clusters associated with angiogenesis, respectively, MCODE-1 and MCODE-5. The top 3 enriched terms of MCODE-1 were “Notch signaling pathway (GO:0007219)”, “Notch signaling pathway(mmu04330)” and “Delta-Notch signaling pathway” (**Figure 6C**). The notch signaling pathway is involved in vascular regeneration in the previous studies (19), and also in models of ischemic brain injury (20, 21). The genes involved in MCODE-5 were mainly enriched in “vascular process in circulatory system”, “circulatory system process” and “regulation of angiogenesis”(**Figure 6D**). These enrichment results suggested that the Itgb2^+^ sc_S3 microglia subcluster was involved in the angiogenesis.

### Neurological recovery as the main role of the sc_S3 on day 7 after tMCAO

**Figure 7:**
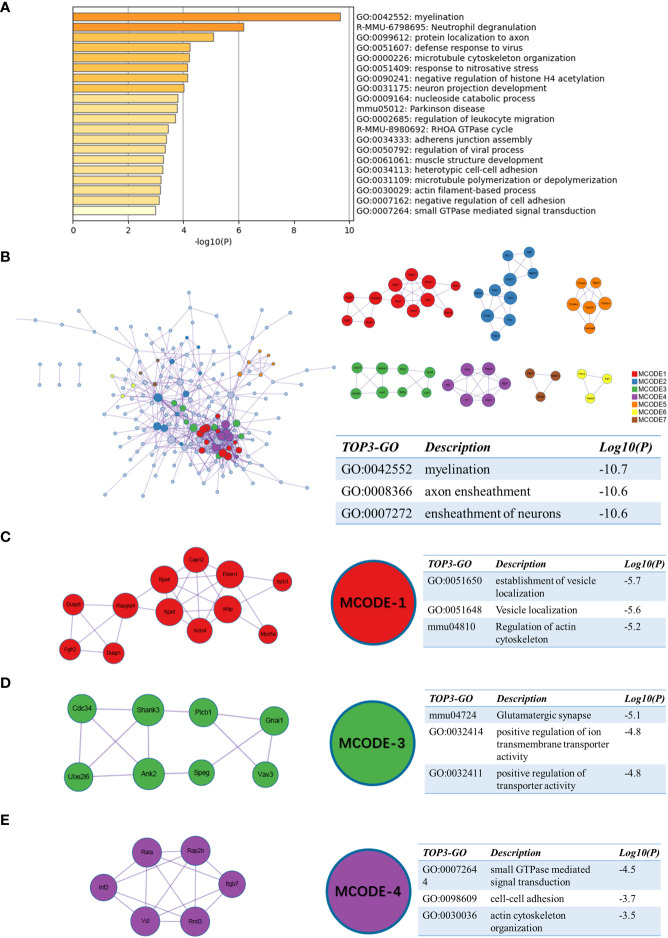
Functional enrichment of the sc_S3 on day 7 after tMCAO. **(A)** The top 20 enriched terms of the sc_S3 on day 7 by Metascape, colored by p-values. **(B)** Protein-protein interaction network and all MCODE components identified of the sc_S3 on day 7. **(C–E)** Representative MCODEs with correlation to enrichment analysis of the sc_S3.

For sc_S3, we performed the same enrichment analysis as the previous one. Among the enriched terms, the representative terms were “myelination”, “protein localization to axon” and “neuron projection development” (**Figure 7A**). This suggested that the cells of sc_S3 might have a neurological recovery function. The PPI network enriched terms like “myelination”, “ensheathment of neurons” and “axon ensheathment” (**Figure 7B**). In the MCODE analysis, the regulation of actin cytoskeleton was involved in both MCODE-1 and 4, a function that was closely related to the neurological recovery and “myelination” that was previously enriched (**Figures 7C, E**). The terms enriched to “Glutamatergic synapse”, “positive regulation of ion transmembrane transporter activity” and “positive regulation of transporter activity” in MCODE-3 are then directly related to synaptic function (**Figure 7D**). In summary, these results indicated that Itgb2^+^ sc_S3 might primarily be responsible for synaptic myelin formation, which might be an important cluster for neurological recovery after ischemic brain injury.

### Functions of Itgb2 in various microglia subclusters

**Figure 8:**
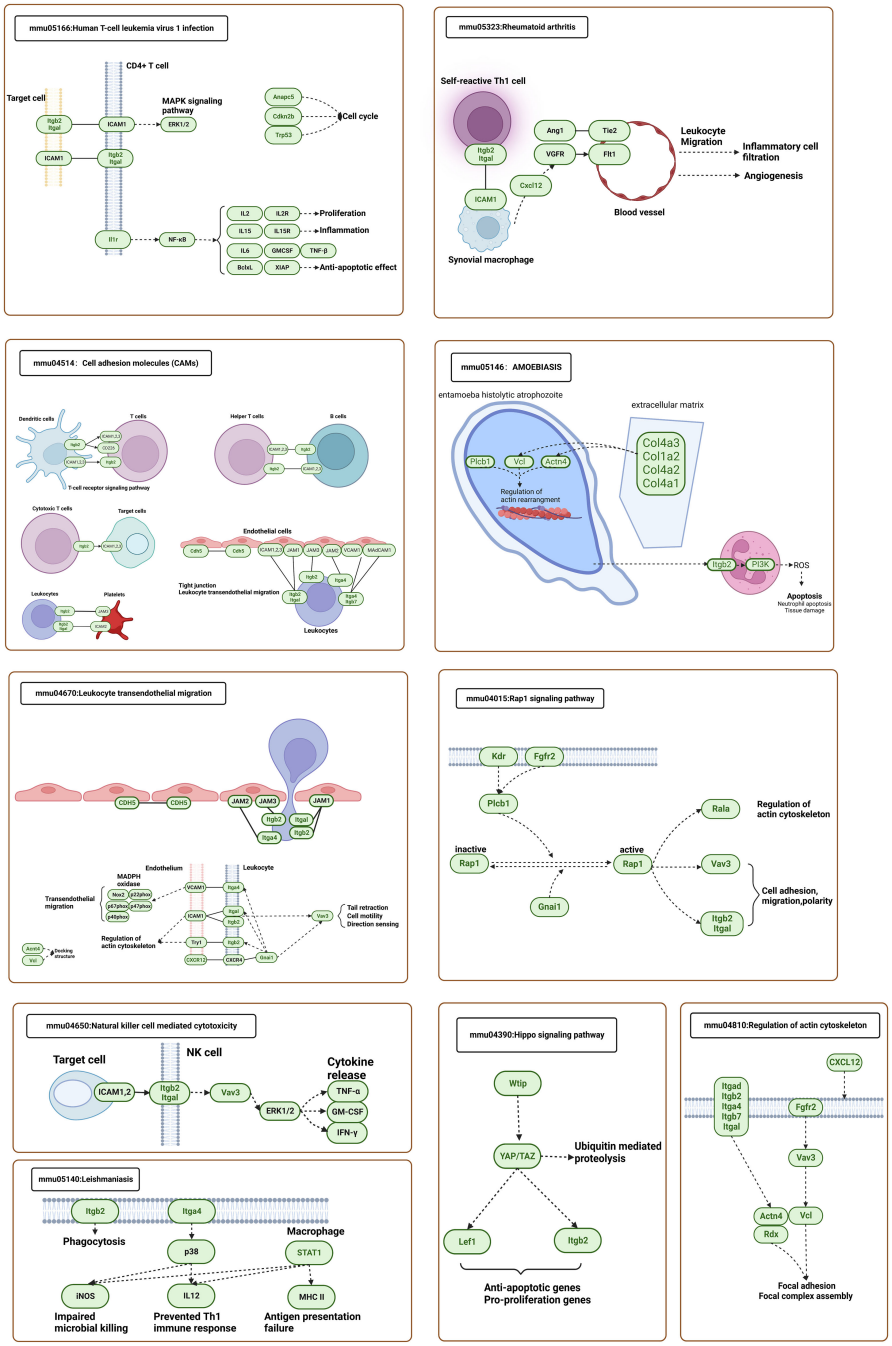
KEGG analysis of the representative MCODEs together with Itgb2 in sc_S1,3,4,5.

To understand the association between Itgb2 and the genes in the representative MCODE of sc_S1, sc_S3, sc_S4, and sc_S5, we grouped Itgb2 and genes in the representative MCODE to perform KEGG analysis, respectively. The following 10 KEGG pathways (**Figure 8**) were screened, which were based on the frequency of KEGG repeats enriched in each cluster. The aim was to allow us to observe and study how Itgb2 works with differential genes in each cluster to function correspondingly at different time points. For example, in “Leishmaniasis” and “Leukocyte Transendothelial Migration”, Itgb2 acted on NADPH oxidase through phagocytosis and calcium signaling pathways, leading to an impaired oxidative burst. This suggested a possible molecular mechanism for the involvement of Itgb2 in the oxidative phosphorylation process of sc_S5 day 1. Similarly, in KEGG of “ Rheumatoid Arthritis”, we found that although Itgb2 and Cxcl12 do not act directly, they can cooperate in the activation of immune cells and VEGF signaling pathway to promote the inflammatory cells migration and angiogenesis. This result was consistent with our enrichment function for sc_S1 on day 3, suggesting one of the ways in which ITGB2 was involved in regulating angiogenesis. The “Regulation of Actin Cytoskeleton” enriched in sc_S3 was also enriched in KEGG of Itgb2, clearly describing the role of Itgb2 as a membrane protein in this pathway. Taken together, we found that Itgb2 function was related to energy metabolism, angiogenesis and cytoskeleton regulation on 1, 3 and 7 days after tMCAO, respectively.

## Discussion

In this study, we provided a source of a single-cell RNA dataset of cerebral ischemic reperfusion injury at different stages. Our results found that the microglia were the most evident increased cell population. We analyzed the cellular interactions between microglia-immune cells and immune cells-neuron. In the analysis of microglia-immune cells receptor-ligand pairs, we found that microglia are major in triggering the immune response after tMCAO modeling. In the analysis of immune cells-neuron, the number of receptor-ligand pairs was largest in microglia-neuron than in the rest of the immune cells. The existence of different functions was found in the conventional microglia subclusters. Further dynamic analysis of microglia by scSTAR showed that the majority of increased microglia distributed in the right upper branches of the trajectory map at 1, 3, and 7 days after tMCAO, compared with the sham group, which microglia is relatively distributed in the branches of trajectory map. Based on our previous transcriptomic and proteomic results, Then, 44 differential expressed genes both in our previous transcriptomic and proteomic results were analyzed through Network Analyzer and Itgb2, the most important gene in topological features of biological function, was selected for further scSTAR analysis. Our further analysis found that the Itgb2^+^ microglia subcluster was mainly related to energetic metabolism and cell cycle, angiogenesis, and participated in neuronal myelination and repair at 1, 3 and 7 days after tMCAO, respectively. Our results suggested that Itgb2^+^ microglia act as a versatile immunoregulating subcluster during the different pathophysiological processes of cerebral ischemic reperfusion injury, which still needs further study to investigate the potential mechanism.

In the current study, we depicted single-cell transcriptional profiles of brain cells at different stages after cerebral ischemic reperfusion injury. The activation of brain-resident immune cells and recruitment of peripheral immune cells at different time points represented the neuroinflammation after cerebral ischemia, which was consistent with previous studies (22). Consistent with previous studies (7, 8), we showed a temporal composition of inflammatory cells after tMCAO, with an increase in the percentage of microglia, monocytes, NK cells and lymphocytes. Specifically, an increased number of microglia and infiltrating immune cells predominated after tMCAO. In our results, the percentage of microglia was the highest increased inflammatory or immune cell population, which was consistent with Guo’s results (8), and microglia have shown polarization and differentiation in two different progression trajectories at 24 hours after MCAO. Many previous studies have reported differently activated microglia. M1 or M2 microglia which normally play a proinflammatory or reparative effect respectively could produce complex immunomodulation in cerebral ischemic-reperfusion injury (23). However, previous studies also indicate that two subclusters of microglia are too crude to exhibit the versatile roles of microglia in cerebral stroke. The advent of single-cell technology provides clear evidence that microglia in the living brain do not polarize into either of these two categories and typically express both M1 and M2 markers. Current subclusters for microglia are described using as many layers of complexity as possible before reaching a consensus and always placing them in a species and spatio-temporal context, by using the term “state” for the description of microglia (24). In our results, subclusters were defined by the function of the clusters at different time points. More recently, two groups reported distinct subclusters of microglia based on the tMCAO mice model.

Compare with their analytic strategy, which both adopted traditional single-cell sequence analysis methods, we selected scSTAR (12) to further unveil the different effects of microglia, which take advantage of the dynamic variation of genes to reflect the capricious pathophysiological process during cerebral reperfusion injury. In conventional scRNA-seq analysis strategies, the variations of interest may be masked by interferences e.g., cell type/subtype heterogeneities may be strong sources of interferences for the variations associated with specific gene function dynamics. scSTAR is designed to extract the variations of interest no matter their amplitudes, which is implemented by constructing real-virtual cell pairs. As a supervised algorithm, scSTAR enables us to precisely target specific gene function dynamics during the cause of tMCAO at 1, 3, 7 days and sham. Based on scSTAR analysis, we found the molecular dynamics of microglia subclusters that changed at different time points in the tMCAO group compared to the sham group and explored the specific functions of Itgb2^+^ microglia clusters at different time points. Dynamic analysis by microglia showed that the trajectory after tMCAO was significantly different from that of the sham group. In the trajectory analysis, we showed that the microglia showed a process of aggregation to the left branch and then reverting to the sham group expression trend. This suggested that we might have a distinct cluster of cells functioning in the early pathogenesis and recovery period. In the trajectory analysis we showed that the microglia exhibited a process of aggregation to the left branch first and then reversion to sham group expression. Compared with previous studies, our results showed a more comprehensive view to understand the variation of microglia at different stages, which may facilitate understanding the biological function of microglia during different phases of stroke. Then, according to our previous omics data from the tMCAO mice model, we found that Itgb2 might play an important role in cerebral ischemia. Itgb2, a member of the integrin family, is involved in the activation of microglia after hypoxia-ischemia in the mouse brain, and Itgb2 receptor blockade or knockout decreased hypoxia-ischemia injury in the first 12 hours (25). Our scSTAR analysis results showed that the different subclusters of Itgb2^+^ microglia may perform different functions on 1,3 and 7 days after tMCAO.

At 1 day after tMCAO, our enrichment analysis and MCODE analysis of sc_S4 and sc_S5 cells representing Itgb2^+^ on day 1 after tMCAO showed that Itgb2^+^ microglia clusters representing the early stroke phase were undergoing rapid proliferation and differentiation (26, 27), and intense energy metabolism (28). These alterations caused early inflammatory activation with microglia and exacerbated the immune response and led to secondary neuronal damage (26, 28, 29). To clarify the function of Itgb2 in MCODE analysis of sc_S4 and sc_S5, we performed KEGG analysis of Itgb2 together with the proteins in PPI of MCODE. In KEGG, we found that Itgb2 was involved in the oxidative phosphorylation process. The involvement of Itgb2 in promoting cancer cell proliferation in cancer by mediating the oxidation of NADH in the mitochondrial oxidative phosphorylation system has also been demonstrated in existing studies (30). And the genes (Ndufb2, Ndufa1, Ndufa3, Ndufc1, and Ndufv3) that we enriched in the MCODE-1 of sc_S5 were all from ubiquinone oxidoreductase subunits. Microglia of Itgb2 were enriched on “Leukocyte Transendothelial Migration” and “Cell Adhesion Molecules” ITGB2/IACM-1 interaction can be seen in KEGG. Intercellular cell adhesion molecule 1 (ICAM-1), as a ligand for ITGB2, is upregulated in endothelial cells in response to muscle ischemia (31). These demonstrated that the genes we found through MCODE were involved in early mitochondrial energy metabolism. It provided a direction for the following research on molecular targets.

Blood-brain barrier (BBB) dysfunction begins with an ischemic episode and worsens with persistent hypoperfusion. Improving early BBB dysfunction can provide substantial protection and improve long-term functional outcomes (32, 33). Recent studies have also demonstrated the role of microglia in vascular repair after injury. Two mechanisms proposed being through mechanical stretching and P2RY12 signaling (34). As Itgb2^+^ sc_S1 on postoperative day 3, the enrichment function mainly reflected vascular regeneration and blood-brain barrier repair. In the MCODE analysis, MCODE-1 was enriched in the Notch signaling pathway, and the existing studies show that downregulation of the Notch signaling pathway inhibits apoptosis of endothelial progenitor cells in mice with ischemic stroke (35, 36). Kdr in MCODE-5 drives meningeal vascular regeneration during head injury (37). In KEGG analysis of Itgb2 together with the proteins in PPI of MCODE, it was demonstrated that endothelial progenitor cell transplantation can promote neovascularization in ischemic tissues through ITGB2/IACM-1 interaction (31, 38). And we speculated this might regulate the neovascularization process in endothelial cells. M2 microglia can protect the CNS, possibly by secreting exosomes that promote angiogenesis (39). We speculated that the microglia of Itgb2 may regulate the neovascularization process in endothelial cells. Therefore, we revealed the gene regulatory network activities of Itgb2^+^ sc_S1, which may involve in hematopoietic reconstitution and damaged blood-brain barrier repair on day 3.

Intracellularly, integrins were linked to the actin cytoskeleton and signaling systems. Integrins regulate synapse formation and maturation, in concert with glial signals, regulate postsynaptic strength by controlling neurotransmitter receptor dynamics and alter dendritic spine shape by triggering actin remodeling (40). It has been reported that Itgb1, which is also in the integrin family, promotes oligodendrocyte regeneration and myelin regeneration in the chronic phase of stroke by affecting the phenotype of microglia (10). Similarly in our functional enrichment and MCODE analyses, Itgb2^+^ sc_S3 microglia enriched to myelin and axon ensheathment regeneration for the chronic phase (7 day after tMCAO), which managed neural repair and functional recovery. The three major enriched MCODEs of differential gene networks were also associated with synaptic and cytoskeletal proteins. The results of KEGG and MCODE analysis were consistent, both associated with the “Regulation of Actin Cytoskeleton” that regulate myelination and axon ensheathment thereby participating in later neural repair. All the above results suggested that different Itgb2^+^ clusters played corresponding effects in different stages of stroke and provided a direction for exploration in our next work.

In conclusion, our study maps a dynamic global single-cell RNA sequencing of brain cells after cerebral ischemic reperfusion injury, broadening our understanding of the heterogeneity of brain cells and the unique gene expression patterns of cell types in cerebral stroke. Furthermore, we also identified Itgb2^+^ microglia, a versatile microglia subcluster, which may participate in different microglia functions at different stages. However, these in silico putative results still need further study to investigate the related molecules, pathways and the specific microglia subcluster.

## Data availability statement

The raw and processed data from single-cell sequencing in this study have been deposited with the Gene Expression Omnibus under accession number GSE227651.

## Ethics statement

The animal study was reviewed and approved by Southern Medical University Administrative Panel on Laboratory Animal Care.

## Author contributions

FZ: Conceptualization; Formal Analysis; Investigation; Methodology; Writing - original draft. JC: Conceptualization; Formal Analysis; Investigation; Methodology; Writing - original draft. ZH: Conceptualization; Formal Analysis; Investigation; Methodology; Writing - original draft. YL: Formal Analysis; Investigation; Methodology. JH: Formal Analysis; Investigation; Methodology. ZQ: Supervision; Funding acquisition; Project administration. XZ: Supervision; Instructing and designing the analysis procedure; Funding acquisition. TT: Supervision; Funding acquisition; Project administration; Writing—review and editing. All authors contributed to the article and approved the submitted version.

## Supplementary material

Supplementary Figure 1 Receptor-ligand pairs in microglia-immune cells as well as immune cells-neuron. **(A)** Heatmap showing the detailed number of receptor-ligand pairs in microglia-immune cells and immune cells-neuron. **(B)** Interaction between cells is shown by the chord-chart. The thickness of the arrow indicates the amount of the number of receptor-ligand pairs.

Supplementary Figure 2 The specific receptor-ligand pairs between microglia and immune cells.

Supplementary Figure 3 The specific receptor-ligand pairs between immune cells and neuron.

Supplementary Figure 4 Conventional scRNA-seq analysis of microglia. **(A)** Bubble map of functional enrichment analysis of microglia subclusters. Colors represent significant differences(p ≤ 0.05). **(B)** Heatmap of top 5 genes for microglia subcluster by the conventional analysis.
