# Supplementary figures and images for: Single-cell analyses reveal the dynamic functions of Itgb2+ microglia subclusters at different stages of cerebral ischemia-reperfusion injury in transient middle cerebral occlusion mice model
Source: Front Immunol. 2023 Mar 30;14:1114663. doi: 10.3389/fimmu.2023.1114663 (PMC10098327; doi:10.3389/fimmu.2023.1114663)

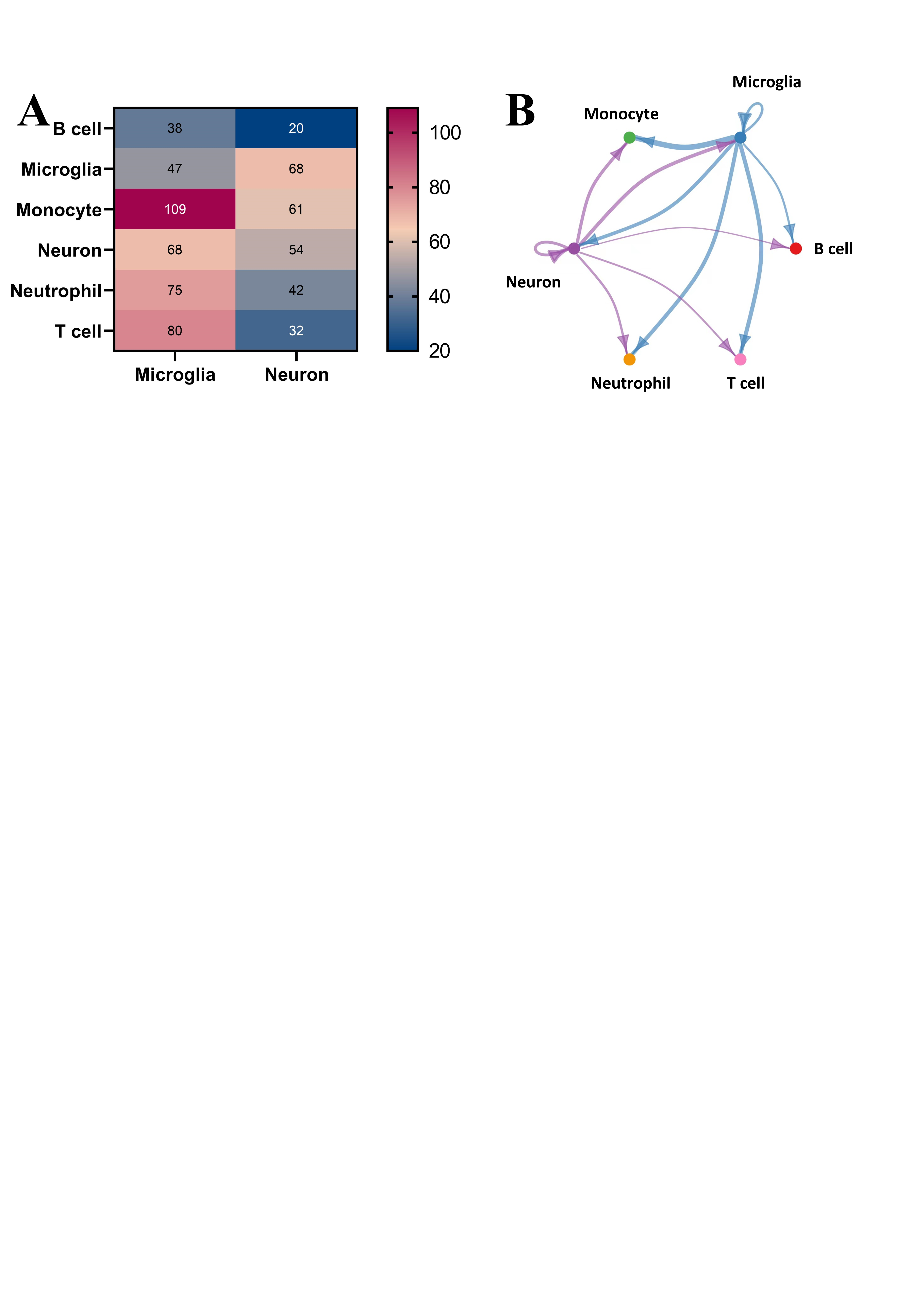

Supplement: Supplementary Figure 1 — Receptor-ligand pairs in microglia-immune cells as well as immune cells-neuron. (A) Heatmap showing the detailed number of receptor-ligand pairs in microglia-immune cells and immune cells-neuron. (B) Interaction between cells is shown by the chord-chart. The thickness of the arrow indicates the amount of the number of receptor-ligand pairs. [file Image_1.tif]

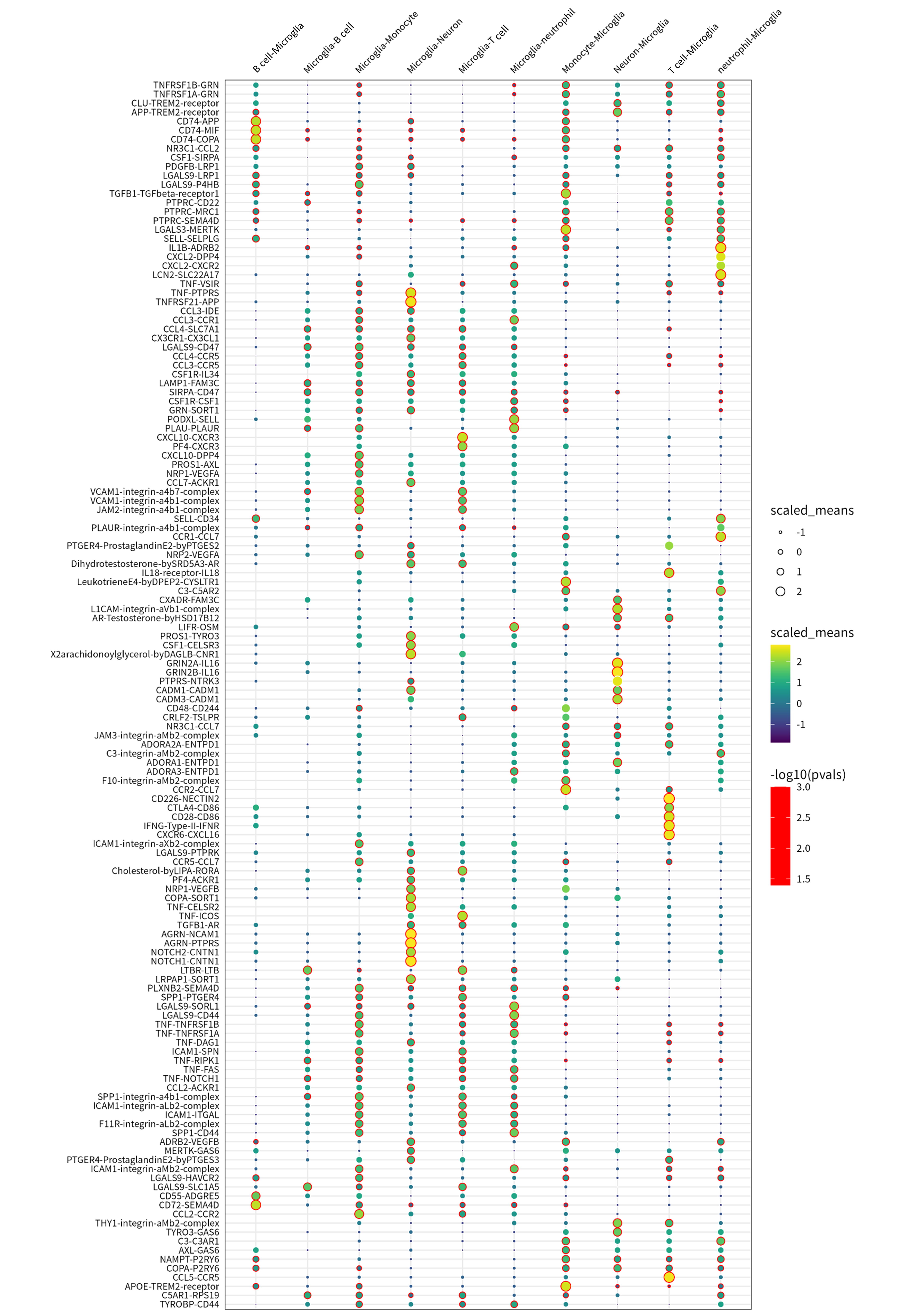

Supplement: Supplementary Figure 2 — The specific receptor-ligand pairs between microglia and immune cells. [file Image_2.tif]

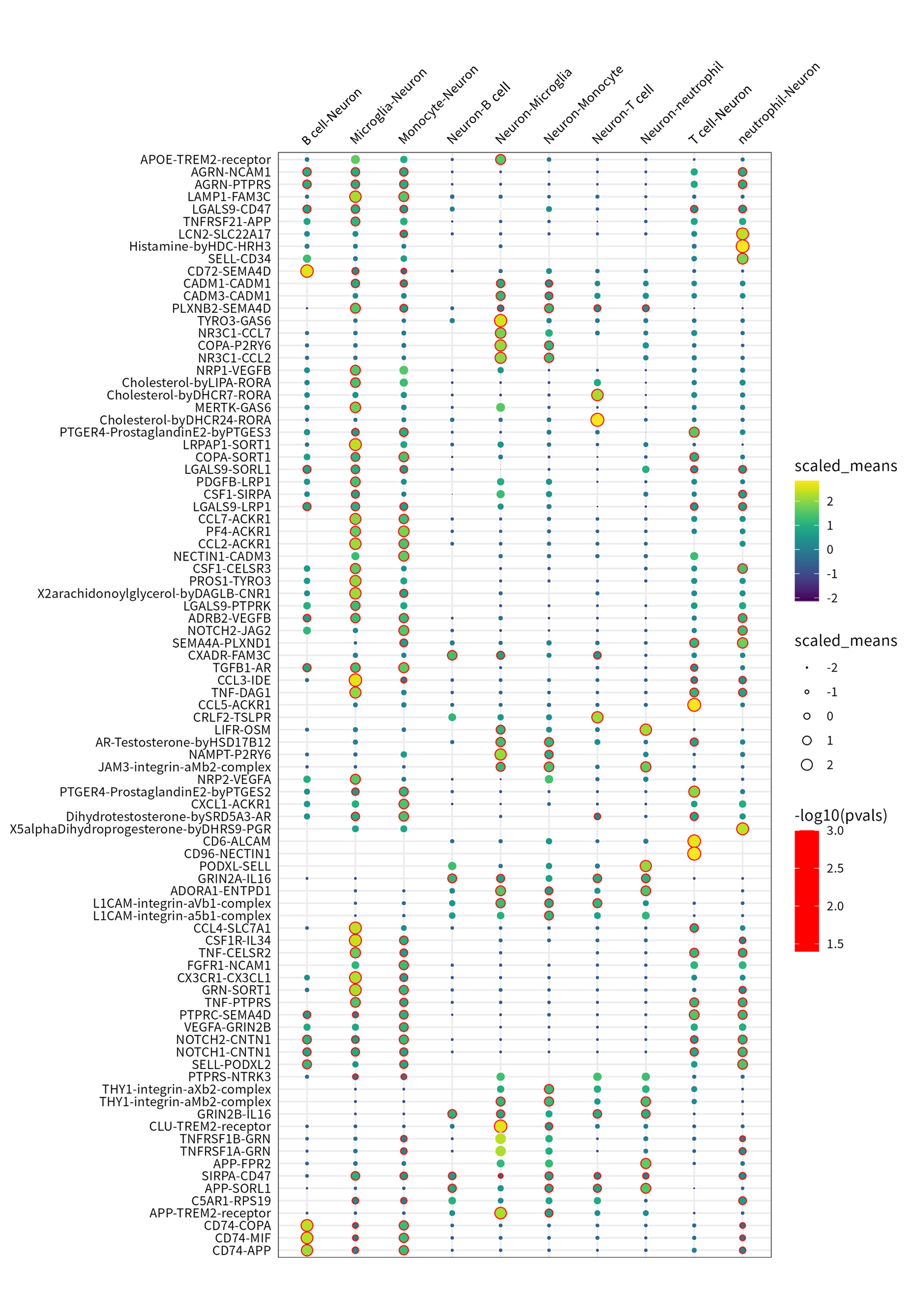

Supplement: Supplementary Figure 3 — The specific receptor-ligand pairs between immune cells and neuron. [file Image_3.tif]

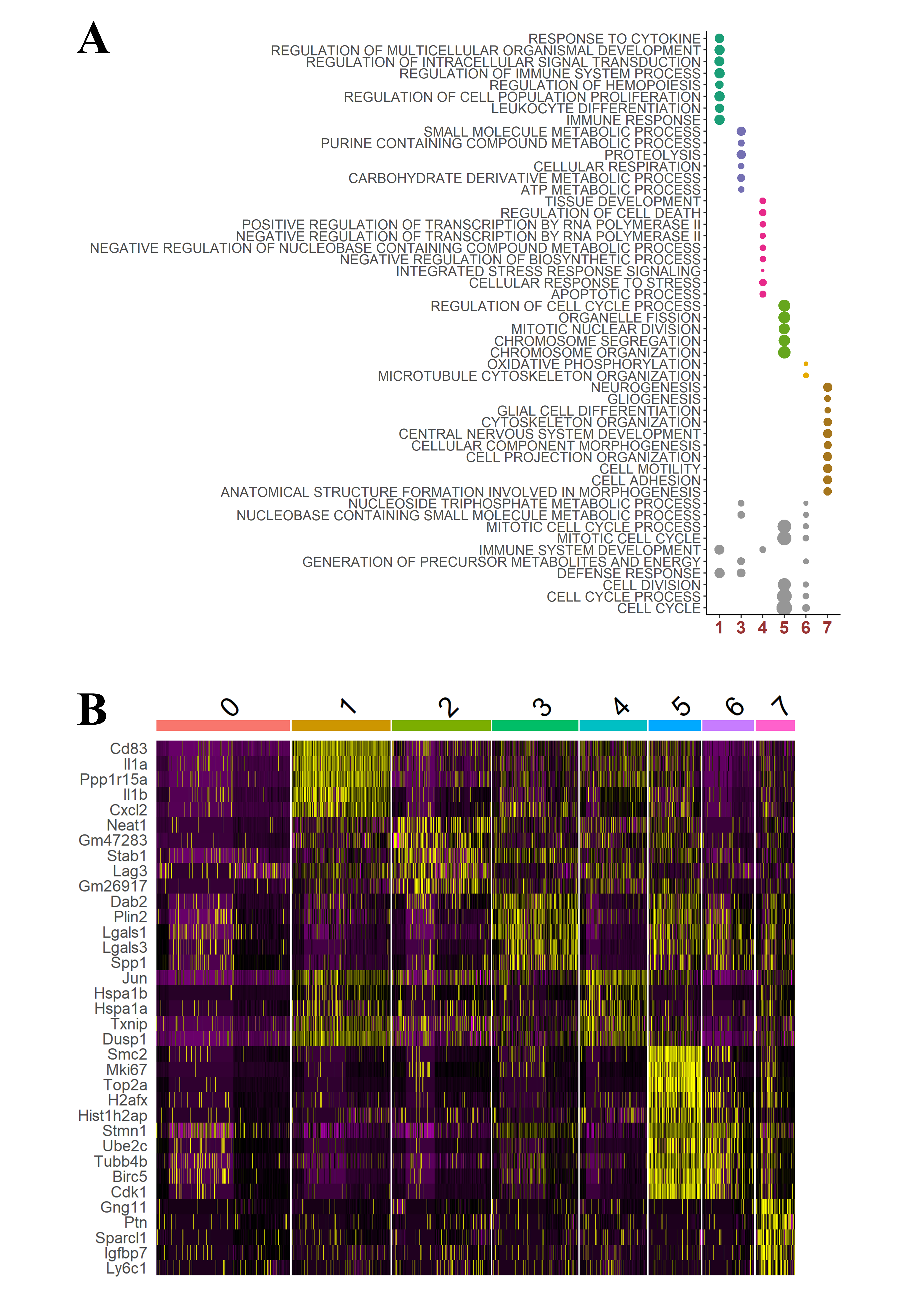

Supplement: Supplementary Figure 4 — Conventional scRNA-seq analysis of microglia. (A) Bubble map of functional enrichment analysis of microglia subclusters. Colors represent significant differences(p ≤ 0.05). (B) Heatmap of top 5 genes for microglia subcluster by the conventional analysis. [file Image_4.tif]
